# Pb_6_Co_9_(TeO_6_)_5_

**DOI:** 10.1107/S1600536812037038

**Published:** 2012-08-31

**Authors:** Christine Artner, Matthias Weil

**Affiliations:** a Institute for Chemical Technologies and Analytics, Division of Structural Chemistry, Vienna University of Technology, Getreidemarkt 9/164-SC, A-1060 Vienna, Austria

## Abstract

Pb_6_Co_9_(TeO_6_)_5_, hexa­lead(II) nona­cobalt(II) penta­tellur­ate(VI), is isotypic with its nickel(II) analogue. The asymmetric unit contains two Pb atoms (site symmetries .2., ..2), four Co atoms (..2, ..2, 3.., 3.2) two Te atoms (..2, 3..) and six O atoms (all in general positions), with the Te and Co sites in octa­hedral coordination environments. The crystal structure can be subdivided into two types of layers parallel to (001). The first layer at *z* ≃ 0.25 is made up of edge-sharing [CoO_6_] and [TeO_6_] octa­hedra, with 1/6 of the octa­hedral holes not occupied. The second layer, situated at *z* ≃ 0, consist of an alternating arrangement of Pb^II^ atoms and of double octa­hedra that are made up from face-sharing [CoO_6_] and [TeO_6_] octa­hedra. The two types of layers are linked together through corner-sharing of [CoO_6_] and [TeO_6_] octa­hedra. The Pb^II^ atoms are situated in the cavities of the framework and are stereochemically active with one-sided [4]- and [6]-coordinations, respectively.

## Related literature

For the isotypic nickel analogue, see: Wedel *et al.* (1998 ▶). Reviews on the crystal chemistry of oxotellurates(VI) and of the geometry of [Co^II^O_6_] polyhedra are given by Levason (1997 ▶) and Wildner (1992 ▶), respectively. For Pb_5_TeO_8_, see: Artner & Weil (2012 ▶). For the bond-valence method, see: Brown (2002 ▶).

## Experimental

### 

#### Crystal data

Pb_6_Co_9_(TeO_6_)_5_
*M* *_r_* = 2891.51
Hexagonal, 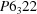

*a* = 10.3915 (1) Å
*c* = 13.6273 (2) Å
*V* = 1274.37 (3) Å^3^
*Z* = 2
Mo *K*α radiation
μ = 50.89 mm^−1^
*T* = 293 K
0.07 × 0.06 × 0.05 mm

#### Data collection

Bruker APEXII CCD diffractometer
Absorption correction: numerical (*HABITUS*; Herrendorf, 1997 ▶) *T*_min_ = 0.123, *T*_max_ = 0.200
45686 measured reflections
2262 independent reflections
1908 reflections with *I* > 2σ(*I*)
*R*_int_ = 0.068

#### Refinement

*R*[*F* ^2^ > 2σ(*F* ^2^)] = 0.025
*wR*(*F* ^2^) = 0.056
*S* = 1.09
2262 reflections
81 parameters
Δρ_max_ = 2.96 e Å^−3^
Δρ_min_ = −2.59 e Å^−3^
Absolute structure: Flack (1983 ▶), 882 Friedel pairs
Flack parameter: 0.134 (10)

### 

Data collection: *APEX2* (Bruker, 2004 ▶); cell refinement: *SAINT* (Bruker, 2004 ▶); data reduction: *SAINT*; program(s) used to solve structure: *SHELXS97* (Sheldrick, 2008 ▶); program(s) used to refine structure: *SHELXL97* (Sheldrick, 2008 ▶); molecular graphics: *ATOMS for Windows* (Dowty, 2006 ▶); software used to prepare material for publication: *publCIF* (Westrip, 2010 ▶).

## Supplementary Material

Crystal structure: contains datablock(s) I, global. DOI: 10.1107/S1600536812037038/br2208sup1.cif

Structure factors: contains datablock(s) I. DOI: 10.1107/S1600536812037038/br2208Isup2.hkl

Additional supplementary materials:  crystallographic information; 3D view; checkCIF report

## Figures and Tables

**Table 1:** Selected bond lengths (Å)

Te1—O2	1.906 (5)
Te1—O6	1.991 (4)
Te2—O5	1.917 (6)
Te2—O3	1.937 (7)
Te2—O1^i^	1.939 (5)
Co1—O5^ii^	2.004 (6)
Co1—O6	2.262 (5)
Co2—O2	2.090 (6)
Co2—O3^iii^	2.090 (8)
Co2—O1	2.108 (6)
Co3—O3	2.107 (4)
Co4—O2	2.067 (7)
Co4—O1^iv^	2.071 (4)
Co4—O5^v^	2.116 (7)

Symmetry codes: (i) 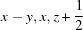; (ii) 
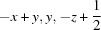
; (iii) 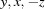; (iv) 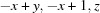; (v) 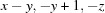.

**Fig. 1.**
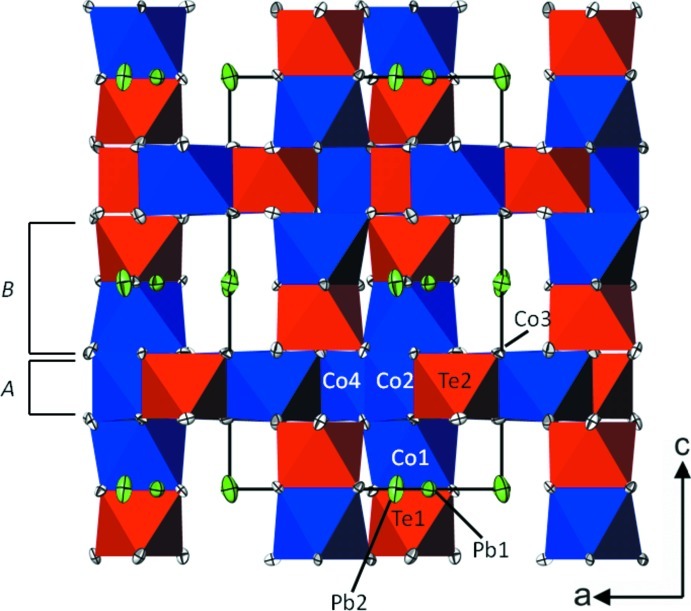
The crystal structure of Pb6Co9(TeO6)5 in a projection along [010]. Displacement ellipsoids are drawn at the 90% probability level. Letters A and B indicate the two types of layers present in the structure.

**Fig. 2.**
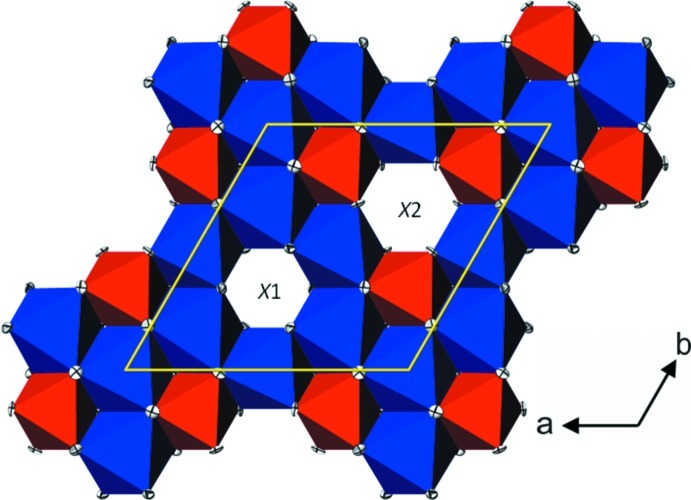
Layer A (situated approximately at z≈ 1/4) in the crystal structure of Pb6Co9(TeO6)5. Colour code and probability of the displacement parameters as in Fig. 1.

**Fig. 3.**
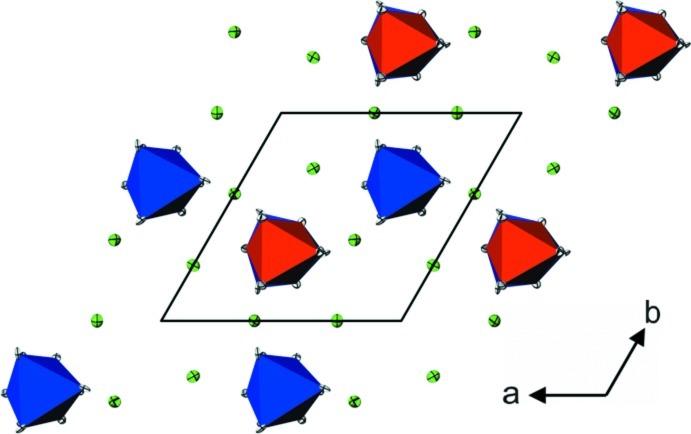
Layer B (situated approximately at z≈ 0) in the crystal structure of Pb6Co9(TeO6)5. Colour code and probability of the displacement parameters as in Fig. 1.

**Fig. 4.**
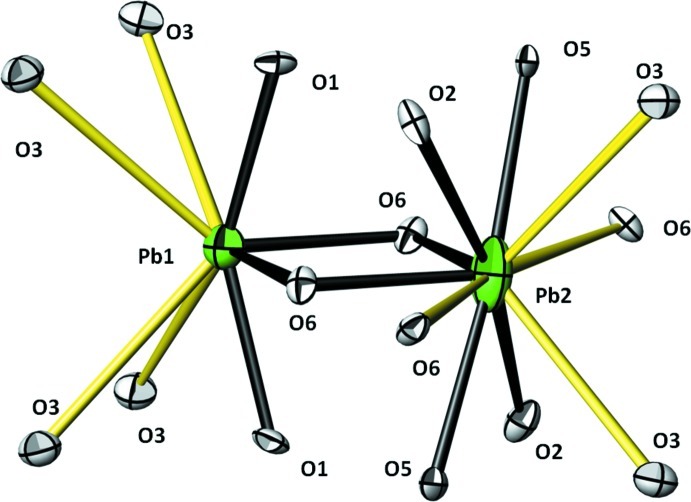
The coordination spheres around the two lead atoms, considering bond lengths up to 3.5 Å; short Pb—O distances < 2.75 Å are given in black, emphasizing the one-sided [4]-coordination for Pb1 and [6]-coordination for Pb2. Longer bonds augmenting the coordination spheres are given in yellow. Probability of the displacement parameters as in Fig. 1.

## supplementary crystallographic information

### Comment

Single crystals of the title compound, Pb_6_Co_9_(TeO_6_)_5_, were serendipitously obtained as a minority phase during phase formation studies in the system Pb^II^/Co^II^/Te^VI^/O intended on crystal growth of cubic Pb_2_CoTeO_6_.

The crystal structure of Pb_6_Co_9_(TeO_6_)_5_ is isotypic with its nickel analogue (Wedel *et al.*, 1998). The two Te(VI) and the four Co(II) atoms are in slightly distorted octahedral coordination environments with mean bond lengths of *¯d*(Te—O) = 1.940 Å and *¯d*(Co—O) = 2.105 Å, both in good agreement with literature data for oxotellurates (Levason, 1997) and for [CoO_6_] octahedra (Wildner, 1992). The two lead(II) atoms exhibit coordination numbers of four and six. The crresponding Pb—O, Te—O and *M*—O (*M* = Co, Ni) bond lengths are very similar in the two isotypic structures.

The crystal structure of Pb_6_Co_9_(TeO_6_)_5_ can be described in terms of (001) layers *A* at *z*≈ 0.25 and *B* at *z*≈ 0 that stack alternately along [100] (Fig. 1). In layer *A* [TeO_6_] and [CoO_6_] octahedra share edges with 1/6 of the octahedral holes at the 2*c* and 2*d* positions, both with site symmetry 3.2, not occupied. The corresponding vacancies, denominated as *X*1 at the 2*d* position and as *X*2 at the 2*c* position, have different sizes. *X*1 has a diagonal diameter of 4.1076 (8) Å whereas *X*2 is somewhat larger with a diagonal diameter of 4.3258 (8) Å. This difference might be correlated with the size of the surrounding octahedra. Whereas the smaller *X*1 vacancy is encircled by a ring of six [CoO_6_] octahedra, the larger *X*2 is encircled by a ring of three [CoO_6_] and three slightly smaller [TeO_6_] octahedra (Fig. 2). Layer *B* consists of double octahedra that are made up from face-sharing [CoO_6_] and [TeO_6_] octahedra, and by surrounding lead(II) atoms (Fig. 3). Adjacent *A* and *B* layers are linked together above and below the *X*1 and *X*2 vacancies through corner-sharing of [CoO_6_] and [TeO_6_] octahedra.

The resulting [Co_9_Te_5_O_30_]^12-^ framework anion leaves space for the stereochemically active lead(II) cations. The oxygen coordination of the two Pb^2+^ cations is one-sided, with a [4]-coordination for Pb1 and a [6]-coordination for Pb2, if only Pb—O distances less than 2.75 Å are taken into account. The two cations share a common edge (O6—O6') with the lone pair electrons *E* pointing towards opposite directions. However, a bond valence calculation (Brown, 2002) shows a significant contribution of the four additional Pb—O distances for each of the two Pb atoms if interactions up to 3.5 Å are considered. Inclusion of these bonds increases the bond valence sum at Pb1 from 1.61 valence units (vu) to 1.83 vu and at Pb2 from 1.64 to 1.96 vu. The bond valence sum at O3 is also raised from 1.60 to 1.80 vu. Therefore the overall coordination of Pb1 might be described as [4 + 4] and that of Pb2 as [6 + 4] (Fig. 4).

### Experimental

1.281 (5.7 mmol) PbO, 0.216 g (2.9 mmol) CoO and 0.914 g (5.7 mmol) TeO_2_ were mixed and thoroughly ground and heated in an alumina crucible under atmospheric conditions during 6 h to 1023 K and held at that temperature for 48 h. Then the furnace was shut-off. Several crystal phases could be identified from the cooled reaction mixture by single-crystal diffraction: Dark blue isometric crystals of Pb_2_CoTeO_6_, dark-red (nearly black) block-like crystals of Pb_5_TeO_8_ (Artner & Weil, 2012), colourless crystals of α-Al_2_O_3_ and dark red crystals of Pb_6_Co_9_(TeO_6_)_5_ with a block-like shape.

### Refinement

The highest remaining electron density was found 1.49 Å from atom Pb1 and the lowest remaining electron density 0.52 Å from atom Pb2. The refined Flack parameter indicates racemic twinning with an approximate ratio of 1:6 for the twin components.

### Crystal data

**Table:**

Pb_6_Co_9_(TeO_6_)_5_	*D*_x_ = 7.535 Mg m^−^^3^
*M**_r_* = 2891.51	Mo *K*α radiation, λ = 0.71073 Å
Hexagonal, *P*6_3_22	Cell parameters from 6739 reflections
Hall symbol: P 6c 2c	θ = 2.8–36.8°
*a* = 10.3915 (1) Å	µ = 50.89 mm^−^^1^
*c* = 13.6273 (2) Å	*T* = 293 K
*V* = 1274.37 (3) Å^3^	Parallelepiped, dark red
*Z* = 2	0.07 × 0.06 × 0.05 mm
*F*(000) = 2470	

### Data collection

**Table:**

Bruker APEXII CCD diffractometer	2262 independent reflections
Radiation source: fine-focus sealed tube	1908 reflections with *I* > 2σ(*I*)
Graphite monochromator	*R*_int_ = 0.068
ω and φ scans	θ_max_ = 37.6°, θ_min_ = 2.3°
Absorption correction: numerical (*HABITUS*; Herrendorf, 1997)	*h* = −16→17
*T*_min_ = 0.123, *T*_max_ = 0.200	*k* = −17→17
45686 measured reflections	*l* = −22→23

### Refinement

**Table:**

Refinement on *F*^2^	Secondary atom site location: difference Fourier map
Least-squares matrix: full	*w* = 1/[σ^2^(*F*_o_^2^) + (0.0244*P*)^2^] where *P* = (*F*_o_^2^ + 2*F*_c_^2^)/3
*R*[*F*^2^ > 2σ(*F*^2^)] = 0.025	(Δ/σ)_max_ < 0.001
*wR*(*F*^2^) = 0.056	Δρ_max_ = 2.96 e Å^−^^3^
*S* = 1.09	Δρ_min_ = −2.59 e Å^−^^3^
2262 reflections	Extinction correction: *SHELXL97* (Sheldrick, 2008), Fc^*^=kFc[1+0.001xFc^2^λ^3^/sin(2θ)]^-1/4^
81 parameters	Extinction coefficient: 0.00019 (3)
0 restraints	Absolute structure: Flack (1983), 882 Friedel pairs
Primary atom site location: structure-invariant direct methods	Flack parameter: 0.134 (10)

### Special details

**Table:**

Geometry. All e.s.d.'s (except the e.s.d. in the dihedral angle between two l.s. planes) are estimated using the full covariance matrix. The cell e.s.d.'s are taken into account individually in the estimation of e.s.d.'s in distances, angles and torsion angles; correlations between e.s.d.'s in cell parameters are only used when they are defined by crystal symmetry. An approximate (isotropic) treatment of cell e.s.d.'s is used for estimating e.s.d.'s involving l.s. planes.
Refinement. Refinement of *F*^2^ against ALL reflections. The weighted *R*-factor *wR* and goodness of fit *S* are based on *F*^2^, conventional *R*-factors *R* are based on *F*, with *F* set to zero for negative *F*^2^. The threshold expression of *F*^2^ > σ(*F*^2^) is used only for calculating *R*-factors(gt) *etc*. and is not relevant to the choice of reflections for refinement. *R*-factors based on *F*^2^ are statistically about twice as large as those based on *F*, and *R*- factors based on ALL data will be even larger.

### Fractional atomic coordinates and isotropic or equivalent isotropic displacement parameters (Å^2^)

**Table:**

	*x*	*y*	*z*	*U*_iso_*/*U*_eq_	
Pb1	0.26736 (3)	0.26736 (3)	0.0000	0.01216 (6)	
Pb2	0.38848 (3)	1.0000	0.0000	0.01881 (8)	
Te1	0.3333	0.6667	−0.09611 (4)	0.00407 (10)	
Te2	0.16730 (4)	0.33460 (9)	0.2500	0.00433 (8)	
Co1	0.3333	0.6667	0.11832 (9)	0.0066 (2)	
Co2	0.16885 (10)	0.3377 (2)	−0.2500	0.00664 (18)	
Co3	0.0000	0.0000	0.2500	0.0087 (3)	
Co4	0.00992 (19)	0.50496 (10)	−0.2500	0.00566 (18)	
O1	0.3366 (5)	0.3241 (5)	−0.1717 (3)	0.0077 (8)	
O2	0.1726 (7)	0.5050 (6)	−0.1628 (3)	0.0083 (10)	
O3	0.1702 (7)	0.1801 (6)	0.3277 (3)	0.0105 (9)	
O5	0.3239 (8)	0.4818 (6)	0.3295 (3)	0.0076 (10)	
O6	0.3481 (4)	0.5265 (4)	−0.0034 (4)	0.0069 (6)	

### Atomic displacement parameters (Å^2^)

**Table:**

	*U*^11^	*U*^22^	*U*^33^	*U*^12^	*U*^13^	*U*^23^
Pb1	0.01117 (9)	0.01117 (9)	0.01243 (12)	0.00429 (9)	0.00014 (9)	−0.00014 (9)
Pb2	0.01104 (9)	0.01201 (13)	0.03372 (18)	0.00601 (7)	−0.00268 (13)	−0.0054 (3)
Te1	0.00414 (14)	0.00414 (14)	0.0039 (2)	0.00207 (7)	0.000	0.000
Te2	0.00362 (13)	0.00380 (19)	0.00564 (18)	0.00190 (10)	0.0002 (4)	0.000
Co1	0.0066 (3)	0.0066 (3)	0.0066 (5)	0.00332 (16)	0.000	0.000
Co2	0.0057 (3)	0.0069 (5)	0.0077 (4)	0.0035 (2)	0.0006 (10)	0.000
Co3	0.0061 (4)	0.0061 (4)	0.0140 (7)	0.00305 (19)	0.000	0.000
Co4	0.0031 (5)	0.0045 (3)	0.0089 (4)	0.0015 (2)	0.000	−0.0002 (3)
O1	0.011 (2)	0.008 (2)	0.0048 (15)	0.0055 (14)	0.0003 (13)	−0.0022 (13)
O2	0.007 (2)	0.006 (2)	0.012 (2)	0.0022 (17)	−0.0001 (16)	−0.0034 (15)
O3	0.011 (2)	0.012 (2)	0.0077 (16)	0.0059 (15)	0.0000 (15)	0.0011 (15)
O5	0.004 (2)	0.006 (2)	0.0072 (18)	−0.0011 (18)	−0.0005 (18)	0.0009 (15)
O6	0.0058 (14)	0.0070 (14)	0.0075 (15)	0.0029 (11)	−0.001 (2)	0.002 (2)

### Geometric parameters (Å, º)

**Table:**

Pb1—O6^i^	2.387 (4)	Te2—O3^iii^	1.937 (7)
Pb1—O6	2.387 (4)	Te2—O3	1.937 (7)
Pb1—O1^i^	2.432 (4)	Te2—O1^xii^	1.939 (5)
Pb1—O1	2.432 (4)	Te2—O1^i^	1.939 (5)
Pb1—O3^ii^	3.327 (7)	Co1—O5^x^	2.004 (6)
Pb1—O3^iii^	3.327 (7)	Co1—O5^iii^	2.004 (6)
Pb1—O3^iv^	3.453 (7)	Co1—O5^xiii^	2.004 (6)
Pb1—O3^v^	3.453 (7)	Co1—O6^vi^	2.262 (5)
Pb1—Pb2^vi^	3.5764 (4)	Co1—O6^vii^	2.262 (5)
Pb1—Pb2^vii^	3.5777 (2)	Co1—O6	2.262 (5)
Pb2—O6^viii^	2.420 (3)	Co2—O2^xiv^	2.090 (6)
Pb2—O6^vii^	2.420 (3)	Co2—O2	2.090 (6)
Pb2—O2^ix^	2.726 (5)	Co2—O3^i^	2.090 (8)
Pb2—O2^vi^	2.726 (5)	Co2—O3^iv^	2.090 (8)
Pb2—O5^x^	2.727 (5)	Co2—O1	2.108 (6)
Pb2—O5^xi^	2.727 (5)	Co2—O1^xiv^	2.108 (6)
Pb2—O6^vi^	3.155 (3)	Co3—O3^xv^	2.107 (4)
Pb2—O6^ix^	3.155 (3)	Co3—O3	2.107 (4)
Pb2—O3^xi^	3.230 (4)	Co3—O3^xvi^	2.107 (4)
Pb2—O3^x^	3.230 (4)	Co3—O3^xvii^	2.107 (4)
Te1—O2^vi^	1.906 (5)	Co3—O3^iii^	2.107 (4)
Te1—O2	1.906 (5)	Co3—O3^v^	2.107 (4)
Te1—O2^vii^	1.906 (5)	Co4—O2	2.067 (7)
Te1—O6^vi^	1.991 (4)	Co4—O2^xviii^	2.067 (7)
Te1—O6^vii^	1.991 (4)	Co4—O1^vi^	2.071 (4)
Te1—O6	1.991 (4)	Co4—O1^xiv^	2.071 (4)
Te2—O5	1.917 (6)	Co4—O5^xix^	2.116 (7)
Te2—O5^iii^	1.917 (6)	Co4—O5^iv^	2.116 (7)
			
O6^i^—Pb1—O6	84.58 (16)	O5^iii^—Te2—O1^xii^	85.9 (2)
O6^i^—Pb1—O1^i^	79.26 (18)	O3^iii^—Te2—O1^xii^	87.0 (2)
O6—Pb1—O1^i^	77.79 (18)	O3—Te2—O1^xii^	93.7 (2)
O6^i^—Pb1—O1	77.79 (18)	O5—Te2—O1^i^	85.9 (2)
O6—Pb1—O1	79.26 (18)	O5^iii^—Te2—O1^i^	93.3 (2)
O1^i^—Pb1—O1	148.79 (19)	O3^iii^—Te2—O1^i^	93.7 (2)
O6^i^—Pb1—O3^ii^	95.87 (13)	O3—Te2—O1^i^	87.0 (2)
O6—Pb1—O3^ii^	133.98 (17)	O1^xii^—Te2—O1^i^	179.0 (3)
O1^i^—Pb1—O3^ii^	147.68 (15)	O5^x^—Co1—O5^iii^	108.14 (14)
O1—Pb1—O3^ii^	56.26 (15)	O5^x^—Co1—O5^xiii^	108.14 (14)
O6^i^—Pb1—O3^iii^	133.98 (17)	O5^iii^—Co1—O5^xiii^	108.14 (14)
O6—Pb1—O3^iii^	95.87 (13)	O5^x^—Co1—O6^vi^	87.99 (18)
O1^i^—Pb1—O3^iii^	56.26 (15)	O5^iii^—Co1—O6^vi^	85.39 (19)
O1—Pb1—O3^iii^	147.68 (15)	O5^xiii^—Co1—O6^vi^	153.55 (18)
O3^ii^—Pb1—O3^iii^	114.8 (2)	O5^x^—Co1—O6^vii^	85.39 (19)
O6^i^—Pb1—O3^iv^	138.20 (17)	O5^iii^—Co1—O6^vii^	153.55 (18)
O6—Pb1—O3^iv^	95.05 (13)	O5^xiii^—Co1—O6^vii^	87.99 (18)
O1^i^—Pb1—O3^iv^	141.63 (15)	O6^vi^—Co1—O6^vii^	72.17 (17)
O1—Pb1—O3^iv^	61.26 (14)	O5^x^—Co1—O6	153.55 (18)
O3^ii^—Pb1—O3^iv^	55.44 (13)	O5^iii^—Co1—O6	87.99 (18)
O3^iii^—Pb1—O3^iv^	87.73 (10)	O5^xiii^—Co1—O6	85.39 (18)
O6^i^—Pb1—O3^v^	95.05 (13)	O6^vi^—Co1—O6	72.17 (17)
O6—Pb1—O3^v^	138.20 (17)	O6^vii^—Co1—O6	72.17 (17)
O1^i^—Pb1—O3^v^	61.26 (14)	O2^xiv^—Co2—O2	87.8 (3)
O1—Pb1—O3^v^	141.63 (15)	O2^xiv^—Co2—O3^i^	92.52 (17)
O3^ii^—Pb1—O3^v^	87.73 (10)	O2—Co2—O3^i^	174.4 (3)
O3^iii^—Pb1—O3^v^	55.44 (13)	O2^xiv^—Co2—O3^iv^	174.4 (3)
O3^iv^—Pb1—O3^v^	111.4 (2)	O2—Co2—O3^iv^	92.52 (17)
O6^viii^—Pb2—O6^vii^	83.17 (17)	O3^i^—Co2—O3^iv^	87.7 (2)
O6^viii^—Pb2—O2^ix^	61.34 (16)	O2^xiv^—Co2—O1	89.3 (2)
O6^vii^—Pb2—O2^ix^	107.91 (19)	O2—Co2—O1	95.5 (2)
O6^viii^—Pb2—O2^vi^	107.91 (19)	O3^i^—Co2—O1	78.9 (2)
O6^vii^—Pb2—O2^vi^	61.34 (16)	O3^iv^—Co2—O1	96.2 (2)
O2^ix^—Pb2—O2^vi^	166.8 (3)	O2^xiv^—Co2—O1^xiv^	95.5 (2)
O6^viii^—Pb2—O5^x^	106.77 (19)	O2—Co2—O1^xiv^	89.3 (2)
O6^vii^—Pb2—O5^x^	68.25 (16)	O3^i^—Co2—O1^xiv^	96.2 (2)
O2^ix^—Pb2—O5^x^	66.27 (12)	O3^iv^—Co2—O1^xiv^	78.9 (2)
O2^vi^—Pb2—O5^x^	112.94 (13)	O1—Co2—O1^xiv^	173.3 (3)
O6^viii^—Pb2—O5^xi^	68.25 (16)	O3^xv^—Co3—O3	175.2 (5)
O6^vii^—Pb2—O5^xi^	106.77 (19)	O3^xv^—Co3—O3^xvi^	79.5 (4)
O2^ix^—Pb2—O5^xi^	112.94 (13)	O3—Co3—O3^xvi^	96.96 (14)
O2^vi^—Pb2—O5^xi^	66.27 (12)	O3^xv^—Co3—O3^xvii^	86.8 (4)
O5^x^—Pb2—O5^xi^	173.7 (3)	O3—Co3—O3^xvii^	96.96 (14)
O6^viii^—Pb2—O6^vi^	138.39 (2)	O3^xvi^—Co3—O3^xvii^	96.96 (14)
O6^vii^—Pb2—O6^vi^	55.23 (15)	O3^xv^—Co3—O3^iii^	96.96 (14)
O2^ix^—Pb2—O6^vi^	126.25 (15)	O3—Co3—O3^iii^	79.5 (4)
O2^vi^—Pb2—O6^vi^	55.67 (17)	O3^xvi^—Co3—O3^iii^	86.8 (4)
O5^x^—Pb2—O6^vi^	60.10 (16)	O3^xvii^—Co3—O3^iii^	175.2 (5)
O5^xi^—Pb2—O6^vi^	120.76 (15)	O3^xv^—Co3—O3^v^	96.96 (14)
O6^viii^—Pb2—O6^ix^	55.23 (15)	O3—Co3—O3^v^	86.8 (4)
O6^vii^—Pb2—O6^ix^	138.39 (2)	O3^xvi^—Co3—O3^v^	175.2 (5)
O2^ix^—Pb2—O6^ix^	55.67 (17)	O3^xvii^—Co3—O3^v^	79.5 (4)
O2^vi^—Pb2—O6^ix^	126.25 (15)	O3^iii^—Co3—O3^v^	96.96 (14)
O5^x^—Pb2—O6^ix^	120.76 (15)	O2—Co4—O2^xviii^	89.8 (3)
O5^xi^—Pb2—O6^ix^	60.10 (16)	O2—Co4—O1^vi^	96.9 (2)
O6^vi^—Pb2—O6^ix^	166.38 (13)	O2^xviii^—Co4—O1^vi^	91.0 (2)
O6^viii^—Pb2—O3^xi^	120.59 (18)	O2—Co4—O1^xiv^	91.0 (2)
O6^vii^—Pb2—O3^xi^	121.18 (18)	O2^xviii^—Co4—O1^xiv^	96.9 (2)
O2^ix^—Pb2—O3^xi^	130.9 (2)	O1^vi^—Co4—O1^xiv^	168.8 (4)
O2^vi^—Pb2—O3^xi^	60.21 (13)	O2—Co4—O5^xix^	174.7 (3)
O5^x^—Pb2—O3^xi^	132.1 (2)	O2^xviii^—Co4—O5^xix^	90.92 (16)
O5^xi^—Pb2—O3^xi^	53.44 (14)	O1^vi^—Co4—O5^xix^	77.8 (2)
O6^vi^—Pb2—O3^xi^	86.29 (19)	O1^xiv^—Co4—O5^xix^	94.2 (2)
O6^ix^—Pb2—O3^xi^	84.37 (18)	O2—Co4—O5^iv^	90.92 (16)
O6^viii^—Pb2—O3^x^	121.18 (18)	O2^xviii^—Co4—O5^iv^	174.7 (3)
O6^vii^—Pb2—O3^x^	120.59 (18)	O1^vi^—Co4—O5^iv^	94.2 (2)
O2^ix^—Pb2—O3^x^	60.21 (13)	O1^xiv^—Co4—O5^iv^	77.8 (2)
O2^vi^—Pb2—O3^x^	130.9 (2)	O5^xix^—Co4—O5^iv^	88.9 (3)
O5^x^—Pb2—O3^x^	53.44 (14)	Te2^ii^—O1—Co4^vii^	98.54 (19)
O5^xi^—Pb2—O3^x^	132.1 (2)	Te2^ii^—O1—Co2	96.67 (17)
O6^vi^—Pb2—O3^x^	84.37 (18)	Co4^vii^—O1—Co2	89.3 (2)
O6^ix^—Pb2—O3^x^	86.29 (19)	Te2^ii^—O1—Pb1	116.6 (2)
O3^xi^—Pb2—O3^x^	93.32 (14)	Co4^vii^—O1—Pb1	136.07 (18)
O2^vi^—Te1—O2	99.15 (18)	Co2—O1—Pb1	110.4 (2)
O2^vi^—Te1—O2^vii^	99.15 (18)	Te1—O2—Co4	129.1 (3)
O2—Te1—O2^vii^	99.15 (18)	Te1—O2—Co2	130.4 (4)
O2^vi^—Te1—O6^vi^	90.7 (2)	Co4—O2—Co2	89.87 (19)
O2—Te1—O6^vi^	85.2 (2)	Te1—O2—Pb2^vii^	95.48 (17)
O2^vii^—Te1—O6^vi^	168.4 (2)	Co4—O2—Pb2^vii^	96.4 (2)
O2^vi^—Te1—O6^vii^	85.2 (2)	Co2—O2—Pb2^vii^	111.0 (2)
O2—Te1—O6^vii^	168.4 (2)	Te2—O3—Co2^xx^	97.34 (17)
O2^vii^—Te1—O6^vii^	90.7 (2)	Te2—O3—Co3	96.2 (2)
O6^vi^—Te1—O6^vii^	84.0 (2)	Co2^xx^—O3—Co3	92.8 (3)
O2^vi^—Te1—O6	168.4 (2)	Te2—O5—Co1^xiii^	125.5 (4)
O2—Te1—O6	90.7 (2)	Te2—O5—Co4^xx^	97.7 (2)
O2^vii^—Te1—O6	85.2 (2)	Co1^xiii^—O5—Co4^xx^	120.3 (3)
O6^vi^—Te1—O6	84.0 (2)	Te2—O5—Pb2^xxi^	117.3 (2)
O6^vii^—Te1—O6	84.0 (2)	Co1^xiii^—O5—Pb2^xxi^	97.82 (18)
O5—Te2—O5^iii^	92.5 (3)	Co4^xx^—O5—Pb2^xxi^	95.2 (2)
O5—Te2—O3^iii^	177.8 (2)	Te1—O6—Co1	86.54 (14)
O5^iii^—Te2—O3^iii^	89.6 (2)	Te1—O6—Pb1	136.5 (2)
O5—Te2—O3	89.6 (2)	Co1—O6—Pb1	127.8 (2)
O5^iii^—Te2—O3	177.8 (2)	Te1—O6—Pb2^vi^	103.41 (16)
O3^iii^—Te2—O3	88.2 (2)	Co1—O6—Pb2^vi^	100.34 (16)
O5—Te2—O1^xii^	93.3 (2)	Pb1—O6—Pb2^vi^	96.12 (13)

Symmetry codes: (i) *y*, *x*, −*z*; (ii) *y*, −*x*+*y*, *z*−1/2; (iii) −*x*+*y*, *y*, −*z*+1/2; (iv) *x*−*y*, *x*, *z*−1/2; (v) *x*, *x*−*y*, −*z*+1/2; (vi) −*x*+*y*, −*x*+1, *z*; (vii) −*y*+1, *x*−*y*+1, *z*; (viii) −*x*+1, −*x*+*y*+1, −*z*; (ix) *y*, *x*+1, −*z*; (x) *x*, *x*−*y*+1, −*z*+1/2; (xi) *y*, −*x*+*y*+1, *z*−1/2; (xii) *x*−*y*, *x*, *z*+1/2; (xiii) −*y*+1, −*x*+1, −*z*+1/2; (xiv) −*x*+*y*, *y*, −*z*−1/2; (xv) −*y*, −*x*, −*z*+1/2; (xvi) −*y*, *x*−*y*, *z*; (xvii) −*x*+*y*, −*x*, *z*; (xviii) *x*, *x*−*y*+1, −*z*−1/2; (xix) *x*−*y*, −*y*+1, −*z*; (xx) *y*, −*x*+*y*, *z*+1/2; (xxi) *x*−*y*+1, *x*, *z*+1/2.
